# Influential Factors of Breastfeeding after Assisted Reproduction: A Spanish Cohort

**DOI:** 10.3390/ijerph19052673

**Published:** 2022-02-25

**Authors:** Jorge Diaz Sáez, José Granero-Molina, María M. López-Rodríguez, Longinos Aceituno Velasco, Cayetano Fernández-Sola, José Manuel Hernández-Padilla, Isabel María Fernández-Medina

**Affiliations:** 1 Obstetrics and Gynaecology Service, Hospital “La Inmaculada”, Huércal-Overa, 04120 Almería, Spain; jorgediazsaez@gmail.com (J.D.S.); laceitunov@sego.es (L.A.V.); 2 Department of Nursing, Physiotherapy and Medicine, University of Almería, 04120 Almería, Spain; jgranero@ual.es (J.G.-M.); cfernan@ual.es (C.F.-S.); j.hernandez-padilla@ual.es (J.M.H.-P.); isabel_medina@ual.es (I.M.F.-M.)

**Keywords:** breastfeeding, breastfeeding barriers, caesarean section, lactation, postpartum care, reproductive techniques

## Abstract

The impact of assisted reproduction techniques (ART) when starting to breastfeed is an important issue that has been sparsely addressed in scientific literature and yet has contradictory results. This study aims to determine the relation between the mode of fertilization and breastfeeding by means of a retrospective longitudinal cohort study that included newborns and mothers who gave birth between 2012 and 2019 in a third-level regional hospital. Data were collected from a total of 11,285 women and newborns, of which 302 (2.6%) used ART. Logistic regression was used to establish models that determine the administration of exclusive breastfeeding (BF). Among the 1208 analyzed participants, 30% conceived using fertility treatment. In this group of participants, BF was less prevalent, both in the delivery room (25.8% versus 45.5%; *p* < 0.001) and when discharged from hospital (42.1% versus 57.9%; *p* < 0.001). Healthy newborns and BF in the delivery room were predictors of BF when discharged. On the other hand, the use of ART, an Apgar score lower than 7 at birth, the use of an epidural and a premature or underweight baby are considered negative predictors of exclusive BF when discharged. It is necessary to offer greater support for all mothers regarding BF, especially those who have conceived through ART, even more so in those cases that involve an epidural and/or caesarean section, starting throughout the dilation process.

## 1. Introduction

Breastfeeding (BF) has been shown to be the best food for newborns (NB) with very few exceptions [1]. Its multiple benefits, both for the mother (lower rate of breast and ovarian cancer, lower risk of type 2 diabetes, and postpartum depression) and for the baby (lower risk of obesity, otitis media, malocclusion, asthma, mortality, and greater intelligence quotients), have been demonstrated [2]. In this sense, parents’ knowledge of these benefits has been found to be an essential facilitator of BF [3]. As a result, the WHO/UNICEF Baby-friendly Hospital Initiative (BFHI) establishes as quality criteria that at least 80% of mothers should be offered breastfeeding assistance within the first 6 hours after giving birth, and for at least 75% of them to exclusively breastfeed their NB when discharged from hospital [4].

However, some factors such as labor, caesarean sections, the feeling of having low milk supply (hypogalactia), artificial milk advertisements, and other individual, social and health factors, such as smoking, obesity, and depression, have been associated with breastfeeding abandonment [5]. On the contrary, according to evidence, other factors are considered protectors in BF, such as a high level of education, uncomplicated delivery, intention to breastfeed during pregnancy, support from family and partner, and attending maternal education classes [6,7]. On the other hand, newborns who start breastfeeding early have higher rates of exclusive BF when discharged from hospital and at two months [8,9]. Regarding the positive effect or not of the use of epidural analgesia on subsequent BF, there seems to be no consensus in scientific literature [10]. Something similar occurs with BF in cases where assisted reproduction techniques (ART) have been carried out.

In terms of infertility, 12–18% of the world’s population has infertility problems [11] and approximately half of the couples in developed countries undergo assisted reproduction treatments [12]. Thus, since the birth of Louise Brown in 1978 in England, more than 4 million children have been born through in vitro fertilization (IVF) treatments, resulting in more than 1 million cycles of IVF being performed every year worldwide [13]. In Spain, sterility figures amount to 10–15% of couples at childbearing age, resulting in being the third country in the world that carries out ART (around one million patients) [14]. The latest report drafted in Spain records a 10% increase in ART demand, with IVF being the most used technique. In addition, births delivered by caesarean section count for 31% of total births in Spain [15]. However, despite the increasing number of assisted reproduction, no recent studies have been found that analyze the demographic profile of the mother who is subjected to this type of conception techniques in Spain, nor the impact that these figures have on BF. 

In this context, the impact of ART when starting to breastfeed and throughout is an important issue that has been sparsely addressed in scientific literature and yet has contradictory results [16,17,18,19,20,21,22,23,24,25]. Despite this, in ART cases, the possible effect on the establishment of BF, among immediate intrapartum or postpartum factors, has not yet been defined.

Thus, this study aims to determine the relation between the mode of fertilization and breastfeeding. 

## 2. Materials and Methods

We conducted a retrospective longitudinal cohort study with a sample of women with live births during their hospitalization immediately after giving birth, using vital records and hospital discharge data. This design was used to investigate relationships between maternal–infant demographic and clinical characteristics and exclusive BF when discharged. This study procedure was approved by the provincial bioethics committee and the management of the “La Inmaculada” Hospital in Huércal-Overa (Almería, Spain).

Although the designation of BFHI was not considered for this study, the changes required for the successful implementation of the "ten steps to successful breastfeeding" developed by the WHO/UNICEF [4] were carried out in this institution before the study was conducted, and the maternity care practices related to BF did not change during the study period.

The cohort in this study included babies born at the “La Inmaculada” Hospital in Huércal-Overa (Almería, Spain) between 2012 and 2019 (*n* = 13,471). In order to avoid possible biases, inclusion criteria were used which stated that participants were at least 16 years old and had given birth to one live child during the study period. Therefore, multiple pregnancies were not included. Likewise, NB with congenital defects or any condition in which exclusive BF was contraindicated or not advised was excluded. 

Therefore, from 2012 to 2019 the data of 11,285 women were collected, of which 302 (2.6%) used some type of medical intervention to conceive (IVF, ovulation induction, artificial insemination, and/or intracytoplasmic sperm injection). 

All mothers who conceived through fertility treatments and met the inclusion criteria were invited to participate (*n* = 302). On the other hand, in cases of spontaneous conception that met the inclusion criteria (*n* = 10,983), the data of 906 women and NB were chosen randomly, in a 3:1 ratio with those conceived by ART to avoid type one errors, which would be in line with other studies of this type [20,22]. In order to obtain this ratio, randomization of the spontaneous conception sample (*n* = 10,983) was carried out with 906 participants in the untreated group included in the analysis. Thus, a total of 1208 participants were finally included in the analyses, of which 30% (*n* = 302) conceived with fertility treatments, which we anticipated would provide adequate power to establish relationships between the mode of fertilization and perinatal care practices.

### 2.1. Measurement 

Variables identified in the literature as associated with BF initiation were evaluated for their potential confounding effects:

- Sociodemographic characteristics: Maternal age, level of education (primary or secondary education), and participants’ nationality;
- Obstetric–neonatal characteristics: Mode of conception, mode of birth, gestational age, use of an epidural, previous births, weight at birth, NB health status, 5-minute Apgar score, BF in the delivery room (which occurs in the first two hours after delivery), and feeding behaviors (exclusive BF or not).

For this research, NB were considered healthy if they did not require paediatric assistance or admission to the neonatal unit or neonatal intensive care unit (NICU). Furthermore, any fertility treatment used at the beginning of pregnancy was considered assisted reproduction compared to a spontaneous mode of conception. Thus, the ART group included the use of medication that increases fertility, artificial insemination and/or IVF including or not the use intracytoplasmic sperm injection. Finally, exclusive BF was defined as 100% human milk feeding from birth until hospital discharge.

### 2.2. Data Collection

Demographic and clinical data were electronically abstracted retrospectively from electronic health records and hospital discharge data. All patient identifying data were eliminated before analysis. The data were collected by a research statistician and kept in a limited access secured data storage.

### 2.3. Data Analysis

Statistical analysis was performed with the SPSS v.23 (IBM Corporation, Armonk, NY, USA) tool. The characteristics of the mother and the child, as well as feeding behaviours, were compared according to the mode of conception using the chi-squared test. After the Kolmogorov–Smirnov analysis, the different modes of conception were compared through non-parametric statistics. Data are presented as mean and standard deviation or the number of patients and percentage. A *p*-value below 0.05 was considered significant. In the regression analysis, maternal age was classified as ≤30 or ≥30 years, and the ordinal variables were recoded to binary factors. Logistic regression was used to establish models that determine the administration of exclusive BF (defined as NB who only receive human milk) when discharged from the maternity hospital. Based on previously reported factors associated with the establishment and maintenance of BF, the independent variables were the following: mode of conception, maternal age, level of education, mode of birth, gestational age, use of an epidural, previous births, weight at birth, NB health status (NB were considered healthy if they did not require paediatric assistance or admission in neonatal unit or NICU), Apgar (performed at 5 minutes), participants’ nationality, and BF in the delivery room (which occurs in the first two hours after delivery). Likewise, we selected a parsimonious model in which the co-linearity between the predictors was avoided.

## 3. Results

In general, the participants in this study were Spanish (71.5 %), over 30 years old, and with at least secondary level education. In order to determine the differences between the groups according to the mode of conception, univariate comparisons were made (Table 1).

When comparing the spontaneous conception group, the participants who conceived with ART were mostly first-time mothers, born in Spain, of legal age, and with secondary or higher education. In addition, participants who used ART had significantly higher percentages of caesarean deliveries and epidural use during childbirth, and gave birth after a significantly shorter pregnancy, as well as giving birth to lower weight NB. On the other hand, participants who had not used any treatment to conceive had vaginal birth and gave birth to a healthy NB in significantly higher percentages. Finally, BF was more prevalent among participants who conceived without fertility treatments, both in the delivery room (45.5% vs. 25.8%; *p* < 0.001) and when discharged from hospital (57.9% vs. 42.1%; *p* < 0.001). On the contrary, no statistically significant differences were found between the group without randomly selected treatments and the rest of the cohort members who were not selected for this study (Table 1).

If we exclusively analyze vaginal deliveries (Table 2) comparing the two types of conception (spontaneous and ART), we still find a significantly higher percentage of participants that are of Spanish nationality, over 30 years of age, primiparous and with a medium or higher level of education in the ART group. Similarly, we observed a significantly higher percentage of preterm and underweight NB and epidural use in the ART group. On the contrary, the highest percentages of healthy NB were found in the group without treatment. Regarding BF, when only vaginal deliveries were analyzed, there was a higher percentage of BF when discharged among those who did not undergo fertility treatments.

By means of the multiple regression analysis, we found that healthy NB and BF in the delivery room were predictors of BF when discharged. On the other hand, the use of ART, an Apgar score of less than 7 at birth, the use of an epidural and a premature or underweight baby are considered negative predictors of exclusive BF when discharged. When the sample of women who conceived with ART were analyzed separately, a vaginal delivery and a higher level of studies were associated to a higher possibility of BF when discharged, while premature and underweight NB decreased this probability. Something similar occurs when analyzing the data of those women who conceived by ART and gave birth vaginally. In these cases, the analysis pointed to the NB’s good health as a positive predictor, while low birth weight could risk BF when discharged (Table 3).

## 4. Discussion

This is the first study to investigate the impact of certain intrapartum and immediate postpartum factors on starting to breastfeed in cases of assisted reproduction. Our results suggest that the mode of conception, the use of an epidural during delivery and an early initiation of BF may have an impact on BF when discharged from hospital. In addition, this study is also the first to identify a relation between fertility treatments and the prevalence of BF when discharged from hospital in a sample based on the Spanish population, describing the profile of the mother undergoing this type of treatment in this part of Europe, compared to mothers who conceived spontaneously.

Some previous studies indicate a lower predominance of BF [16,17,18,19], others report that at least the figures for BF when discharged from the hospital are higher in cases treated with ART [20,21], with a better predisposition by these mothers [21]. At the same time, the studies conducted by O’Quinn et al. (2012) [22] and Kermani et al. (2012) [23] state that there are no differences between either type of conception. In turn, there are suggestions that the highest rate of caesarean sections, multiple births, and premature or underweight NB among ART cases [24,25] may influence starting to breastfeed. 

According to the analyzed data in this study, the group of participants who became pregnant through ART were mostly having their first child, born in Spain, of legal age, and with secondary or higher education, and gave birth to a NB with a lower weight and a lower gestational age.

Firstly, the data related to the maternal age of this study coincide with other studies [17,22,26] where the age of the ART group was significantly higher and, furthermore, other research, such as this one, found differences in the nationality of women in both groups [17,20,26]. On the other hand, the level of studies in the ART group in this study was higher, coinciding with the results by Michels et al. (2016) [20] and Barrera et al. (2019) [26]. However, this variable did not obtain significant differences in the studies by Fisher et al. (2013) [17], O’Quinn et al. (2012) [22] and Kermani et al. (2012) [23]. Although the highest incidence of a caesarean section in ART is confirmed today in scientific literature [17,20,26], no research was found to contrast the greater use of an epidural observed in this study in the ART group. Finally, our data showed a greater number of primiparous mothers, preterm NB, and lower birth weight in the ART group, coinciding with Michels et al. (2016) [20] and Barrera et al. (2019) [26]. 

According to our data, BF when discharged from hospital was less prevalent in the ART group. This coincides with the BF discharge data shown by Cromi et al. (2015) [16], Fisher et al. (2013) [17], Wiffen and Fetherston (2016) [18], and Barnes (2013) [19], and differs from the results by Michels et al. (2016) [20], Barrera et al. (2019) [26], and Hammarberg et al. (2011) [21], who obtained higher BF figures upon discharge in the ART group. In the study conducted by O’Quinn et al. (2012) [22], no differences were observed between the groups. When analyzing vaginal births in isolation, we found that, although significant differences continued to appear, they were equated between both groups. As some studies assure [5,6], this fact would highlight the importance of the mode of delivery when initiating BF, caesarean sections being the main barrier for its correct development. 

Regarding the early onset of BF, our results showed a greater incidence in the spontaneous conception group. However, when analyzing vaginal births in isolation, this variable showed no significant differences. Although most of the consulted studies did not measure this variable, our results coincide with those by Cromi et al. (2015) [16], stating that there was no difference from BF during the first hour after delivery according to the mode of conception, using a design controlled by age and maternal parity, type of delivery and gestational age.

By means of the regression analysis, healthy NB and BF in the delivery room were found to be protective factors of BF cessation and positive predictors of BF when discharged from hospital. These data coincide with Ashley’s review [6] on influential factors in the onset of BF in general, which points out the delay in the onset of BF after delivery as one of the hospital practices related to a lower incidence and/or duration of BF. Likewise, coinciding with the results obtained by Fisher et al. (2013) [17], conception through ART was revealed as an obstacle to initiate BF. In addition, an Apgar score of less than 7, the use of an epidural, and a premature or low-weight baby were shown as risk factors of BF cessation and barriers of exclusive BF when discharged. Again, this coincides with Ashley’s review [6] which indicates the use of epidurals, prematurity, and underweight NB as barriers to BF, as they will imply admitting the baby to a hospital ward. On the other hand, the review by French et al. (2016) [10] regarding epidural analgesia is inconclusive in terms of whether or not it influences BF [8]. In contrast to other studies [17], caesarean sections were not revealed to be a negative predictor of BF when discharged from hospital in our analysis of the total sample.

However, in the ART group, and coinciding with Fisher et al. (2013) [17], vaginal deliveries were associated with a higher probability of BF when discharged. Furthermore, the level of studies of the mother was also a positive predictor, coinciding with the study by Hammarberg et al. (2011) [21] which states there is an increase in the risk of BF abandonment in participants without university studies. These data could be related, in turn, to Silva’s review [7] which pointed out that BF is the most sensitive result to prenatal educational strategies, as a higher level of education could facilitate the assimilation of said educational strategies. On the contrary, as in cases of spontaneous conception, prematurity and underweight NB were identified as barriers for BF when discharged. These results coincide with Kermani et al. (2012) [23] where the authors conclude that the most influential factor on the form of feeding is the baby’s weight. In general, these data are broadly consistent with Ashley’s review [6], where it was determined that a high level of studies, vaginal delivery, not separating mother and baby (due to not being premature or underweight), and starting BF in the delivery room are positive predictors of BF when discharged.

Thus, our results indicate that factors, such as conception through ART, use of epidurals, caesarean sections, or altered newborn health, which make BF when discharged difficult, are easily detectable. On the contrary, the importance of beginning to breastfeed in the delivery room is evidenced in our study, which leads us to insist on the desirability of this action. For all these reasons and in view of these results, we believe that greater support is necessary, which, expertly and based on evidence, has an impact on the women who go through these situations in order to establish BF when discharged from hospital. In this sense, it would be advisable to improve the information provided throughout pregnancy when conceived with ART regarding a possible caesarean delivery and the use of the epidural, therefore increasing the possible risks of lower BF that these factors entail.

The data presented in this study provide the first approach of the impact of certain intrapartum and immediate postpartum factors in establishing BF in cases of assisted reproduction. However, there are some possible limitations to this research, as, for example, the non-inclusion of variables related to psychological problems, such as depression or stress [27], and maternal postnatal. In addition, data on women’s intention to breastfeed during pregnancy, attending maternal education classes, or nursing information regarding BF offered during hospitalization were not addressed in this study. Likewise, a BF follow-up consultation after hospital discharge would have significantly completed the results of this study. Finally, our sample obtained a reduced number of conceptions through ART (under 3% of the total sample), compared to the number of spontaneous. Future research could transfer data collection to areas with a larger population.

## 5. Conclusions

In conclusion, our results suggest that the mode of conception, using an epidural during delivery and the early initiation of BF may have an impact on BF when discharged from hospital. In addition, this study is the first to identify the associations between fertility treatments and the prevalence of BF upon discharge in a Spanish sample, describing the profile of the mother undergoing this type of treatment in this part of Europe, compared to those who conceived spontaneously.

## Figures and Tables

**Table 1:** Sociodemographic data of the sample (n = 1208).

		Mode of Conception				χ^2^	*p*	Remainder Cohort N = 10,077		χ^2^	*p*
		Spontaneous *n* = 906 (75%)		ART *n* = 302 (25%)							
		N	%	N	%						
Born in Spain		600	66.2%	264	87.4%	49.943	*p* < 0.001	6855	66.0%	0.012	0.913
Maternal Age	>30	498	55.0%	263	87.1%	100.240	*p* < 0.001	5617	54.1%	0.241	0.623
	<30	408	45.0%	39	12.9%			4762	45.9%		
Secondary education		621	68.5%	252	83.4%	25.093	*p* < 0.001	7227	69.6%	0.466	0.495
Previous parity		488	53.9%	57	18.9%	111.984	*p* < 0.001	5610	54.1%	0.016	0.899
Preterm Birth		44	4.9%	37	12.3%	19.801	*p* < 0.001	381	3.7%	3.232	0.072
Normal delivery		781	86.2%	175	57.9%	109.539	*p* < 0.001	9113	87.8%	1.972	0.160
Epidural		319	35.2%	136	45.0%	9.309	0.002	3864	37.2%	1.457	0.227
Apgar < 7		37	4.1%	18	6.0%	1.835	0.176	317	3.1%	2.907	0.088
Healthy NB		807	89.1%	239	79.1%	19.248	*p* < 0.001	9259	89.2%	0.016	0.899
Underweight		43	4.7%	48	15.9%	40.410	*p* < 0.001	392	3.8%	2.112	0.146
Sex	Female	454	50.1%	161	53.3%	0.929	0.335	5115	49.3%	0.229	0.633
	Male	452	49.9%	141	46.7%			5264	50.7%		
Discharge BF		525	57.9%	127	42.1%	23.033	*p* < 0.001	6136	59.1%	0.474	0.491
BF in Delivery room		412	45.5%	78	25.8%	36.263	*p* < 0.001	4831	46.5%	0.384	0.535
		**Mean**	**SD**	**Mean**	**SD**	**Z**	***p***	**Mean**	**SD**	**Z**	***p***
Maternal age (years)		30.27	5.93	35.44	4.99	−12.822	*p* < 0.001	30.27	5.93	−0.077	0.939
NB Gestational age (weeks)		39.25	1.51	38.66	1.78	−4.558	*p* < 0.001	39.23	1.34	−1.112	0.266
NB weight (grams)		3314.39	461.40	3077.19	562.82	−5.902	*p* < 0.001	3298.44	501.49	−1.593	0.111
Apgar		8.72	1.14	8.60	1.15	−1.188	0.235	8.71	0.98	−1.499	0.134

NB: Newborn; BF: Breastfeeding; Z: U Mann–Whitney; SD: Standard Deviation.

**Table 2:** Sociodemographic and clinical categorical data in vaginal delivery (*n* = 956).

		Mode of Conception					
		Spontaneous *n* = 781		ART *n* = 175		χ^2^	*p*
		N	%	N	%		
Born in Spain		508	65.0%	150	85.7%	28.468	*p* < 0.001
Maternal Age	>30	415	53.1%	151	86.3%	65.043	*p* < 0.001
	<30	366	46.9%	24	13.7%		
Secondary education		529	67.7%	147	84.0%	18.265	*p* < 0.001
Previous parity		435	55.7%	46	26.3%	49.472	*p* < 0.001
Preterm Birth		36	4.6%	20	11.4%	12.055	0.001
Epidural		276	35.3%	96	54.9%	22.912	*p* < 0.001
Apgar < 7		24	3.1%	10	5.7%	2.908	0.088
Healthy NB		704	90.1%	145	82.9%	7.631	0.006
Underweight		31	4.0%	23	13.1%	22.575	*p* < 0.001
Sex	Female	387	49.6%	86	49.1%	0.010	0.922
	Male	394	50.4%	89	50.9%		
Discharge BF		463	59.3%	89	50.9%	4.160	0.041
BF in Delivery room		411	52.6%	78	44.6%	3.711	0.054
		**Mean**	**SD**	**Mean**	**SD**	**Z**	***p***
Maternal age (years)		30.06	5.97	34.87	4.51	−9.951	*p* < 0.001
NB Gestational age (weeks)		39.26	1.33	38.90	1.73	−1.844	0.065
NB weight (grams)		3309.99	491.03	3134.63	529.99	−3.824	*p* < 0.001
Apgar		8.76	0.86	8.62	1.13	−1.369	0.171

ART: Assisted Reproductive Technology; NB: Newborn; BF: Breastfeeding; Z: U Mann–Whitney; SD: Standard Deviation.

**Table 3:** Factors associated with BF when discharged.

	B	Standard Error	*p*	OR	95% C.I. for EXP(B)		R^2^ Cox and Snell
					Lower	Upper	
**Factors Associated with BF when Discharged in the Whole Sample (*n* = 1208)**							
Preterm Birth Yes (Ref) No	−0.901	0.308	0.003	0.406	0.222	0.743	0.092
Epidural Yes (Ref) No	−0.266	0.126	0.034	0.766	0.599	0.980	
Underweight Yes (Ref) No	−0.885	0.294	0.003	0.413	0.232	0.734	
Apgar < 7 Yes (Ref) No	−0.688	0.336	0.040	0.503	0.260	0.971	
Healthy NB Yes (Ref) No	0.415	0.210	0.048	1.515	1.004	2.286	
Conception ART(Ref) Spontaneous	−0.353	0.144	0.014	0.703	0.530	0.931	
BF in Delivery room Yes (Ref) No	0.597	0.127	*p* < 0.001	1.816	1.417	2.328	
**Factors Associated with BF when Discharged in ART (*n* = 302)**							
Preterm Birth Yes (Ref) No	−1.372	0.598	0.022	0.254	0.079	0.819	0.151
Normal Delivery Yes (Ref) No	0.856	0.259	0.001	2.354	1.416	3.914	
Secondary Education Yes (Ref) No	0.803	0.344	0.020	2.233	1.138	4.382	
Underweight Yes (Ref) No	−1.609	0.530	0.002	0.200	0.071	0.566	
**Factors Associated with BF when Discharged in** **Vaginal Births with ART (*n* = 175)**							
Healthy NB Yes (Ref) No	1.041	0.534	0.051	2.833	0.995	8.065	0.135
Low Weight Yes (Ref) No	−2.173	0.789	0.006	0.114	0.024	0.535	

ART: Assisted Reproductive Technology; NB: Newborn; BF: Breastfeeding; OR: odds ratio; CI: Confidence Interval.

## Data Availability

Not applicable.
